# Prescriptions

**Published:** 1897-04

**Authors:** 


A GOOD STIMULATING EXPECTORANT FOR ADULTS.

℞ Apomorph, hydrochloratis . 1 gr.

Syr. ipecacuanhæ ......... 2 drs.

Syr. tolutani .............. 1 oz.

Aquæ dist., q. s. ad ....... 3 ozs.

A teaspoonful five times daily at four hours intervals. Shake.

IODOL IN THE TREATMENT OF ATONIC ULCERS.

The *Presse medicale* gives the following formula for an ointment.

℞ Iodol .................... 2 parts.

Vaseline, Lanoline, } each ......... 10 "

M. To be spread thin on aseptic lint and applied.

IGCONTINENCE OF URINE.

℞ Strychniæ ............... gr. j.
Acidi acetici ............ gtt. ij.
Sacchari albi ............ ʒ ij.
Aquæ destillatæ ......... ℥ ij.
Ft. solutio

M. Sig.: Fifteen to thirty drops for a child six to twelve years old.

*Magendie.*

PNEUMONIA.

℞ Ammonii carbonatis .... gr. iv.
Spts. chloroformi ....... ♏ xx.
Aquæ camphoræ ........ ʒ v.

M. Sig.: To be given every three or four hours- (*When delerfum is present, with small weak, pulse.*)

*Waters.*

